# High-throughput sequencing of IgG B-cell receptors reveals frequent usage of the rearranged IGHV4–28/IGHJ4 gene in primary immune thrombocytopenia

**DOI:** 10.1038/s41598-019-45264-2

**Published:** 2019-06-14

**Authors:** Makoto Hirokawa, Naohito Fujishima, Masaru Togashi, Akiko Saga, Ayumi Omokawa, Tomoo Saga, Yuki Moritoki, Shigeharu Ueki, Naoto Takahashi, Kazutaka Kitaura, Ryuji Suzuki

**Affiliations:** 1 Department of General Internal Medicine and Clinical Laboratory Medicine, Akita University Graduate School of Medicine, Akita, Japan; 2 Division of Blood Transfusion, Akita University Hospital, Akita, Japan; 3 Department of Hematology, Nephrology and Rheumatology, Akita University Graduate School of Medicine, Akita, Japan; 4 Repertoire Genesis Incorporation, Ibaraki, Japan; 5 Department of Rheumatology and Clinical Immunology, Clinical Research Center for Allergy and Rheumatology, Sagamihara National Hospital, Sagamihara, Japan

**Keywords:** Molecular medicine, Medical research

## Abstract

Primary immune thrombocytopenia (ITP) is an acquired form of thrombocytopenia caused by IgG anti-platelet autoantibodies and represents an organ-specific autoimmune disorder. Although the glycoprotein (GP)IIb/IIIa and GPIb/IX have been shown to be targets for autoantibodies, the antigen specificity of autoantibodies is not fully elucidated. To identify the characteristics of IgG B-cell receptor (BCR) repertoires in ITP, we took advantage of adaptor-ligation PCR and high-throughput DNA sequencing methods for analyzing the clone-based repertoires of IgG-expressing peripheral blood B cells. A total of 2,009,943 in-frame and 315,469 unique reads for IGH (immunoglobulin heavy) were obtained from twenty blood samples. Comparison of the IGHV repertoires between patients and controls revealed an increased usage of IGHV4–28 in ITP patients. One hundred eighty-six distinct IGHV4–28-carrying sequences were identified in ITP patients and the majority of these clones used an IGHJ4 segment. The IGHV4–28/IGHJ4-carrying B-cell clones were found in all ITP patients. Oligoclonal expansions of IGHV4–28/IGHJ4-carrying B cells were accompanied by multiple related clones with single amino substitution in the CDR3 region suggesting somatic hypermutation. Taken together, the expansion of IGHV4–28/IGHJ4-carrying IgG-expressing B cells in ITP may be the result of certain antigenic pressure and may provide a clue for the immune pathophysiology of ITP.

## Introduction

Primary immune thrombocytopenia (ITP) is an acquired form of thrombocytopenia caused by anti-platelet autoantibodies. The underlying mechanism is thought to involve the production of IgG autoantibodies specific for platelet membrane antigens, such as glycoprotein (GP)IIb/IIIa and GPIb/IX, although anti-platelet autoantibody testing is less sensitive for the diagnosis^1,2^. The ASH and IWG guidelines for the management of ITP do not recommend routine testing of anti-platelet autoantibodies for the diagnosis of ITP, and thus diagnostic biomarkers for ITP need to be developed^3–5^.

Although the principal pathophysiology of ITP is an IgG-mediated autoimmune disease, the B-cell receptor (BCR) repertoires associated with this disorder are largely unknown. The spleen is generally believed to be the primary site for the activation of T and B cells responsible for autoantibody production in primary ITP^6,7^. Interestingly, however, Kuwana *et al*. found that B cells secreting anti-GPIIb/IIIa or anti GPIb antibodies can be detected in the peripheral blood as well as spleen from primary ITP patients using an enzyme-linked immunospot (ELISPOT) assay^7–9^. In addition, others have reported that antigen-specific IgG-bearing memory B cells can be detected in circulating blood in humans^10^.

High-throughput sequencing of BCR genes have revealed the landscape and longitudinal changes of B-cell repertoires and have identified clonal expansions^11–18^. Recently, Kitaura *et al*. have developed a new BCR repertoire analysis methods comprised of adaptor-ligation polymerase chain reaction (PCR) and next-generation sequencing, which enables the comprehensive quantitative analysis of BCRs at a clonal level^19^. Somatic hypermutation among antibody subclasses can be easily disclosed by this method. Taking advantage of this novel method, we investigated the repertoires of IgG-BCRs of peripheral blood B cells from ITP patients in order to identify the characteristics of IgG-BCR repertoires in this disorder, and were able to find the oligoclonal expansions of IGHV4–28/IGHJ4-carrying IgG-expressing B cells with small clonal sizes.

## Results

### IGHV repertoires of IgG BCRs in primary ITP

**Figure 1:**
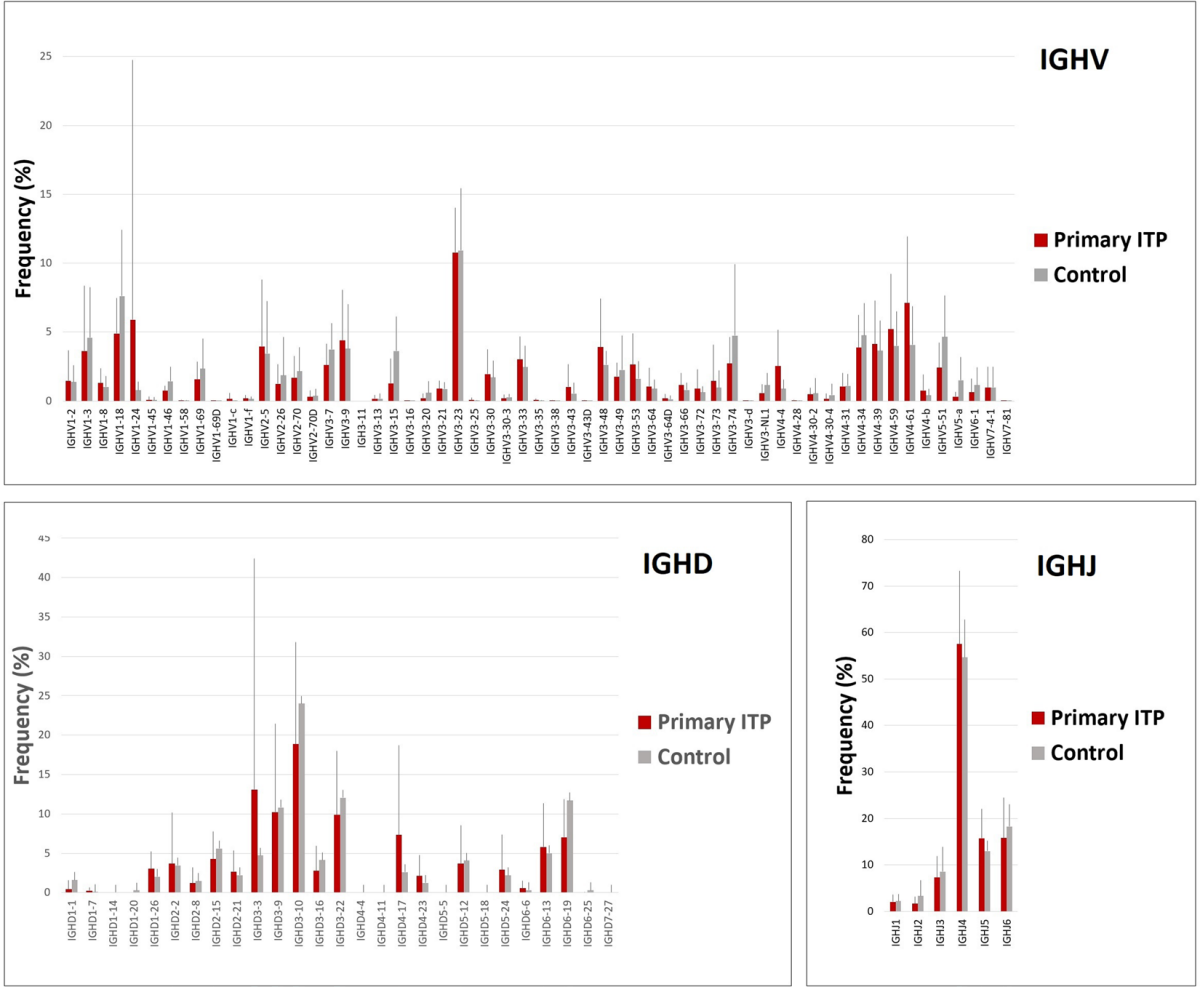
Comparison of the usage of IGHV, IGHD, and IGHJ genes of IgG BCRs. Mean percentage usages of IGHV, IGHD and IGHJ are shown. Bars and error bars indicate mean percentage usage and its standard deviation of eleven ITP patients and nine control donors. The mean values of IGHV1–24 and IGVD3–3 were much higher in ITP than those in control that was due to the presence of one outlier for the ITP cohort. There was no significant difference in the usages of IGHV, IGHD, and IGHJ between the patients and controls except the IGHV4–28 and IGHV3–15 segments.

**Figure 2:**
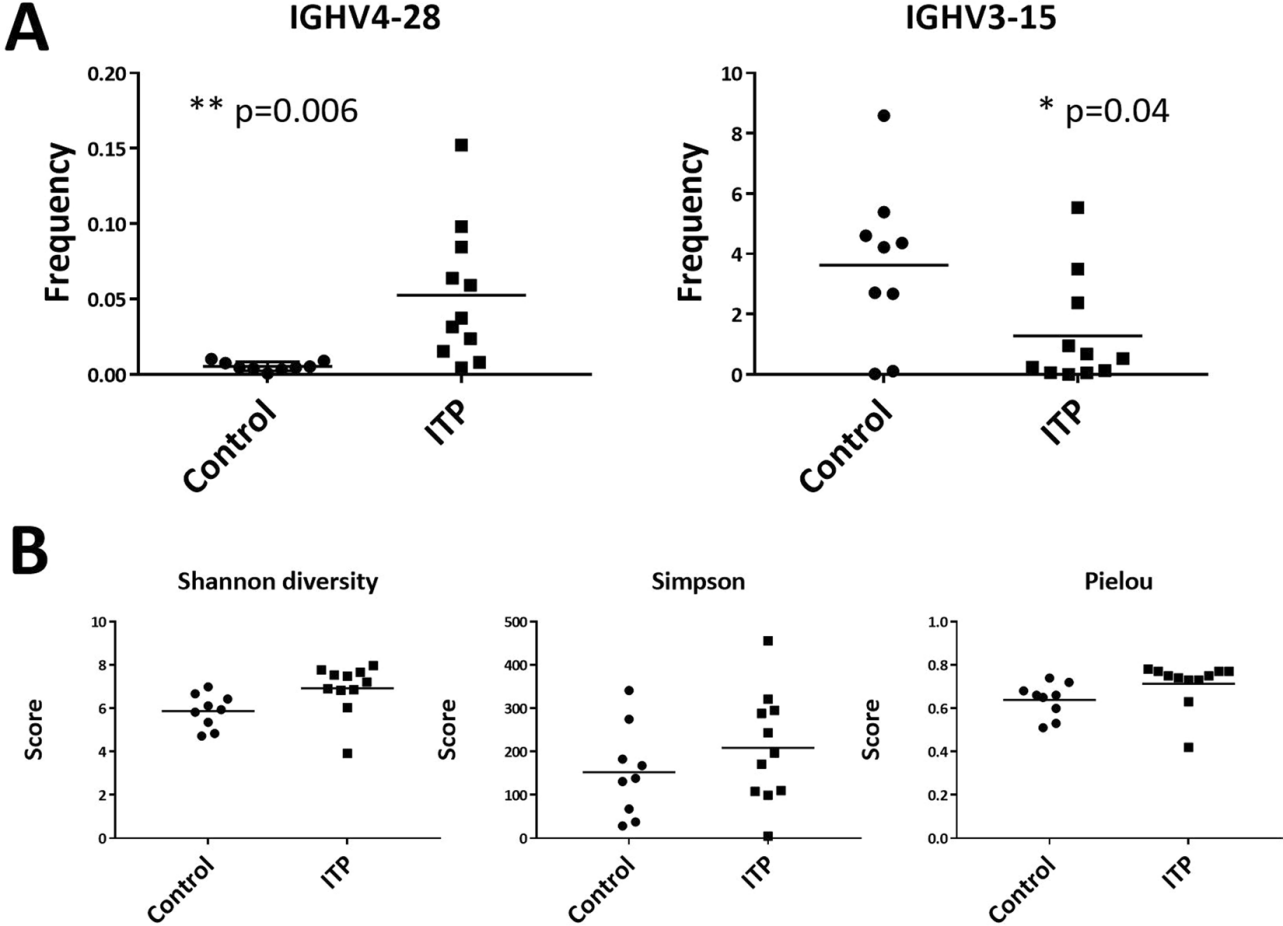
IGHV repertoires and diversity in primary ITP. (**A**) Characteristic IGHV repertoires of IgG BCRs in primary ITP were an increased usage of the IGHV4–28 segment and a decreased usage of the IGHV3–15 segment. (**B**) Diversity indices of Simpson and Pielou were not statistically different between the two groups (p = 0.303 and 0.095, respectively). The Shannon scores were slightly higher in ITP patients (p = 0.031). The total numbers of in-frame reads in ITP and control were 885,793 and 1,124,150, respectively, and the total unique reads in ITP and control were 215,640 and 99,829, respectively. Thus, the richness in B-cell clones was even higher in the ITP group than the control group of this cohort.

A total of 2,009,943 in-frame and 315,469 unique reads were obtained from twenty blood samples, and 29,049 to 160, 013 reads (100,497 reads in average) from each sample. The global usage of IGHV, IGHD, and IGHJ segments were not different between the patients and controls (Fig. 1). Patient characteristics are described in Supplementary Table 1. The mean values of IGHV1–24 and IGVD3–3 were much higher in ITP than those in control and this was the presence of one outlier for the ITP cohort. In this particular ITP patient, the expansion of IGHV1–24-carrying B-cell clones was detected, although its clinical significance was not clear. In other ten ITP patients, the IGHV1–24 subfamily comprised less than 1% of total B-cell repertoire. However, we found significantly increased usage of IGHV4–28 (0.053% vs. 0.005%, p = 0.006) and less usage of IGHV3–15 (1.28% vs. 3.63%, p = 0.04) in ITP patients (Fig. 2A). Diversity indices of Simpson and Pielou were not statistically different between the two groups, but the Shannon scores were slightly higher in ITP patients (Fig. 2B). The total numbers of in-frame reads in ITP and control were similar, but the total unique reads in ITP were higher. Thus, the richness in B-cell clones in the ITP patient cohort might have affected the difference in Shannon diversity scores.

### B-cell clones carrying IGHV4–48 in primary ITP

**Figure 3:**
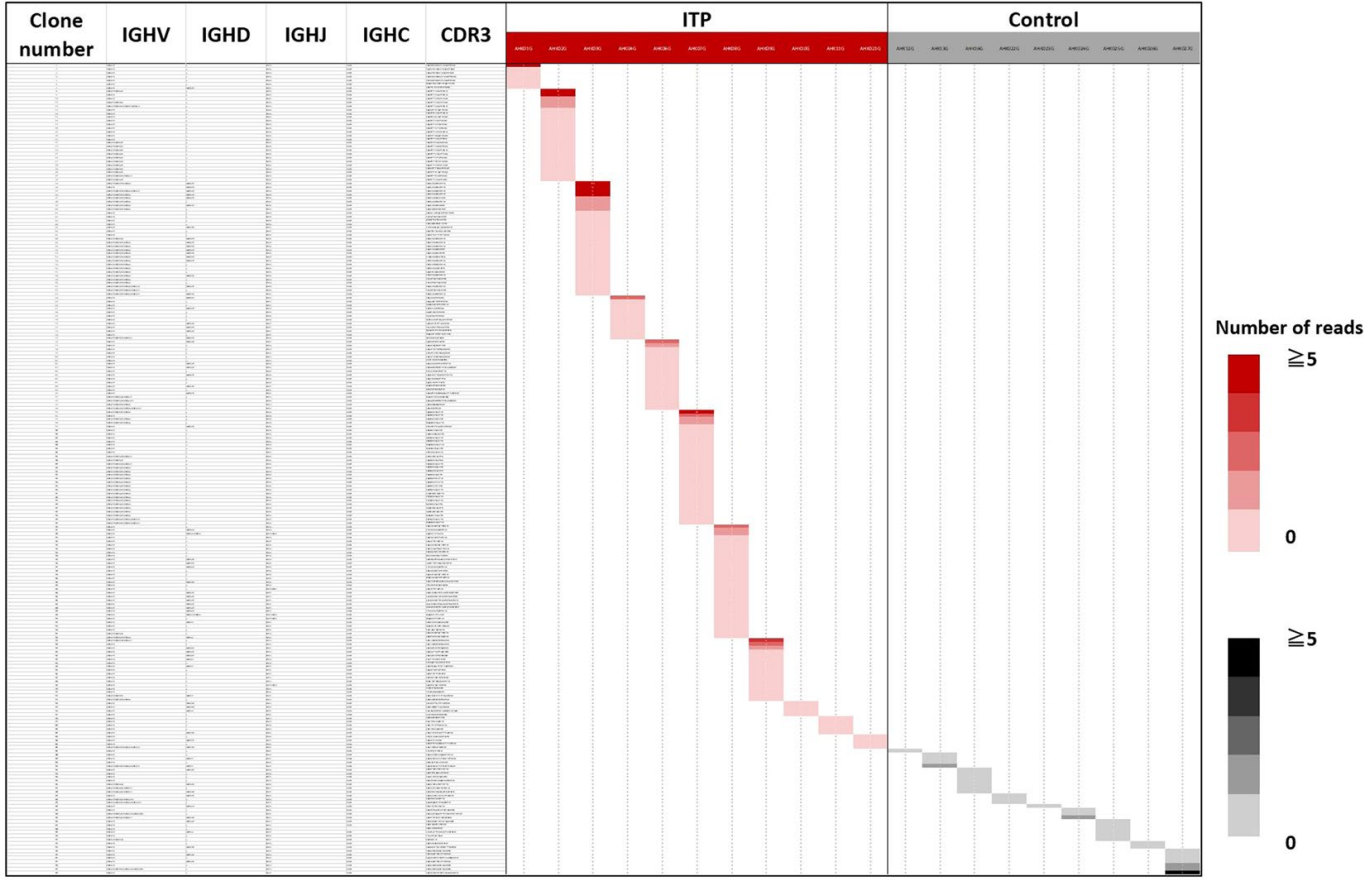
IgG-BCR-bearing B-cell clones carrying IGHV4–28 in primary ITP. Each row indicates a respective B-cell clone and each column on the right-hand side means each individual. The usage of IGH segments and the CDR3 sequences are available on supplementary data (Supplementary dataset for Fig. 3).

There were 186 and 34 distinct IGHV4–28-carrying sequences identified in ITP patients and controls, respectively (Fig. 3). One-hundred twenty-six of 186 IGHV4–28-carrying clones (67.7%) used the IGHJ4 gene segment in patients with ITP, and 9 of 34 IGHV4–28-carrying clones (26.5%) used the IGHJ4 gene segment in control donors. There were neither shared public clones among ITP patients nor between the ITP and the control groups. Because somatic hypermutation in non-CDR3 regions conferred some conflicts on an assignment of IGHV segments in IGHV4 subfamilies, we compared the sequence reads of V, D, J segments and CDR3 of certain clones and considered them as using the same IGHV gene if they shared the same CDR3 sequences and IGHJ genes.

IGHV4–28/IGHJ4-carrying B-cell clones were found in all ITP patients. Among these 126 IGHV4–28/IGHJ4-carrying clones, most B-cell clones used IGHG1 (51.6%) and IGHG2 (44.4%) gene segments as constant regions.

### Deduced amino acid sequences of the CDR3 region of overexpressed IgG-BCR with IGHV4–28

**Figure 4:**
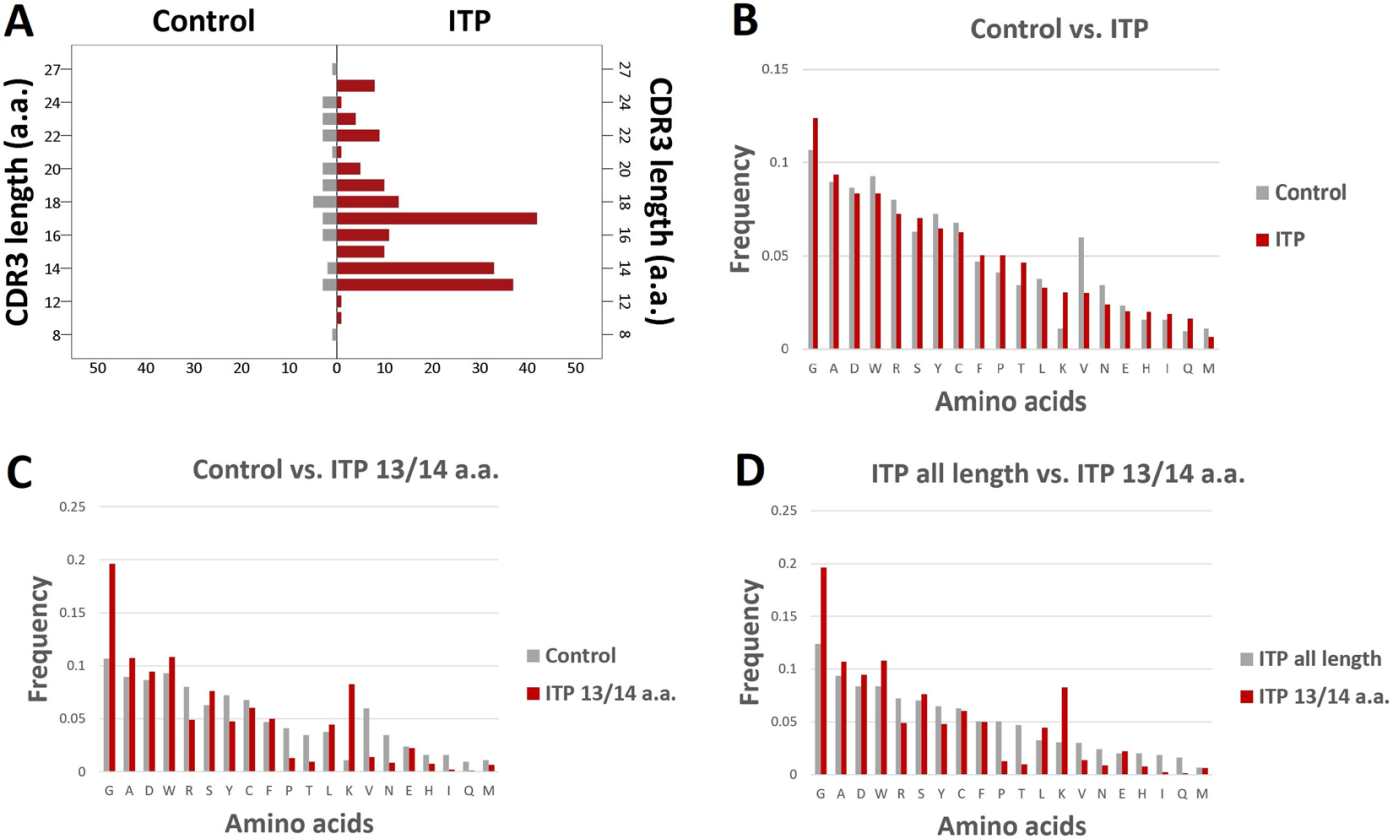
Deduced amino acid sequences of the CDR3 of overexpressed IGHV4–28 IgG-BCR in ITP. (**A**) CDR3 length distribution was skewed with an additional peak at 13 and 14 amino acids in ITP. (**B**) Composition of amino acids in the CDR3 of IGHV4–28-BCRs of all clones from ITP patients was compared with those from control donors. (**C**) Composition of amino acids in the short CDR3 of IGHV4–28-BCRs from ITP patients was compared with those from control donors. (**D**) Composition of amino acids in the short CDR3 of IGHV4–28-BCRs from ITP patients was compared with those of all IGHV4–28-carrying B-cell clones from ITP patients.

We examined the structures of the CDR3 region of IgG-BCRs expressed on IGHV4–28-carrying B-cell clones. The distribution of CDR3 lengths of 186 distinct clones in ITP were compared to that of 34 clones in control donors (Fig. 4A). Previously, Kitaura *et al*. reported that the CDR3 length of all IgG subclasses in healthy individuals showed a Gaussian-like distribution with a median length of 18 amino acids^19^. In the present ITP patient cohort, the CDR3 length distribution was skewed with an additional peak at 13 and 14 amino acids. This was supported by the difference in distribution parameters between control and ITP (supplementary Table 2). Glycine (G) and lysine (K) were more frequently used in these short CDR3 sequences of IGHV4–28-carrying BCRs than those in all IGVH4–28-using BCRs in ITP and controls (Fig. 4B–D, Supplementary Tables 3 and 4). Five IGVH4–28 clones with a 13/14 amino acid length CDR3 were detected in control donors, but lysine was not used in those clones (Supplementary dataset for Fig. 3). There was no apparent sequence homology in the CDR3 region among patients (Table 1).

**Table 1:** Deduced amino acid sequences of CDR3 of over-expressed IgG BCRs using the IGHV4–28 segment.

UPN	IGHV	IGHD	IGHJ	CDR3	Number of reads*
AHK01G	IGHV4–28	x	IGHJ4	CARDMYDCNHVCSGSADYFDQW	6
AHK02G	IGHV4–28	x	IGHJ4	CARIPPTTGTAHYFDQW	5
AHK02G	IGHV4–28	x	IGHJ4	CARIPPTTGTDHYFDQW	2
AHK02G	IGHV4–28,IGHV4–61	x	IGHJ4	CARIPPTTGTAHYFDQW	17
AHK02G	IGHV4–28,IGHV4–61	x	IGHJ4	CARIPPTTGTAHYCDQW	2
AHK02G	IGHV4–28,IGHV4–61,IGHV4–59,IGHV4-4	x	IGHJ4	CARIPPTTGTAHYFDQW	2
AHK03G	IGHV4–28	IGHD6–19	IGHJ4	CARKGSGWDGFDFW	8
AHK03G	IGHV4–28,IGHV4–59,IGHV4-4	IGHD6–19	IGHJ4	CARKGSGWDGFDFW	115
AHK03G	IGHV4–28,IGHV4–59,IGHV4-4	IGHD6–19	IGHJ4	CARKGSGWDGFDFW	5
AHK03G	IGHV4–28,IGHV4–59,IGHV4-4	IGHD6–19	IGHJ4	CARKGSGWDGCDFW	2
AHK03G	IGHV4–28,IGHV4–59,IGHV4-4	IGHD6–19	IGHJ4	CARKGSGWDGFAFW	2
AHK03G	IGHV4–28,IGHV4–59,IGHV4-4,IGHV4–39	IGHD6–19	IGHJ4	CARKGSGWDGFDFW	6
AHK03G	IGHV4–28,IGHV4–59,IGHV4-4	x	IGHJ4	CARKGSGWDGFDFW	2
AHK03G	IGHV4–28,IGHV4–59,IGHV4-4	x	IGHJ4	CARKGRGWDGFDFW	2
AHK04G	IGHV4–28	IGHD4–17	IGHJ4	CARGHGDYEFDHW	3
AHK06G	IGHV4–28	x	IGHJ4	CARDEGWEMPYVYW	2
AHK06G	IGHV4–28	IGHD3–10	IGHJ5	CARRDPTIVRGVFPW	3
AHK07G	IGHV4–28	x	IGHJ4	CAKKGDGSALGYW	3
AHK07G	IGHV4–28,IGHV4–61,IGHV4-4	x	IGHJ4	CAKKGDGSALGYW	20
AHK07G	IGHV4–28,IGHV4–61,IGHV4-4	x	IGHJ4	CAKKGDGSARGYW	2
AHK07G	IGHV4–28,IGHV4–61,IGHV4-4	x	IGHJ4	WAKKGDGSALGYW	2
AHK08G	IGHV4–28	x	IGHJ4	CARGHCRGPNCYSPDFW	3
AHK08G	IGHV4–28	IGHD4–23	IGHJ4	CTRGNYGGNWPPDFW	2
AHK08G	IGHV4–28	IGHD3–3,IGHD3–9	IGHJ5, IGHJ4	CAARLLSSYYVGLW	2
AHK09G	IGHV4–28	x	IGHJ3	CARTGKNSWSRPDALDIW	3
AHK09G	IGHV4–28,IGHV4–34,IGHV4–31	x	IGHJ3	CARTGKNSWSRPDALDIW	4
AHK09G	IGHV4–28	IGHD4–17	IGHJ3	CARGAIYGDYDPNAFDIW	2

*The CDR3 sequences were presented only for IgG-BCRs detected more than once.

### Somatic hypermutation in the CDR3 region of IGHV4–28/IGHJ4 carrying IgG-BCR

To test whether the expansion of IgG BCR using a IGHV4–28 segment was the result of certain antigenic pressures, we analyzed the deduced amino acid sequences of the CDR3 region of all IGHV4–28/IGHJ4 BCR in the same individual. As shown in Table 2, twenty-one distinct B-cell clones with the rearranged IGHV4–28/IGHJ4 gene were identified in this patient (patient number AHK03G), and clone #33 was the most prevalent clone (Table 2). Among B-cell clones with the same CDR sequence, there were several clones with distinct non-CDR3 sequences (clone #34, 48, 33, 36, 38, 35, 63). Fourteen other clones had CDR3 regions with a single amino acid substitution compared to that of clone #33. This oligoclonal expansion of particular B cells with IGHV4–28/IGHJ4-carrying IgG-BCR and the presence of multiple related clones with a single amino substitution in the CDR3 region were observed in two other patients AHK07G and AHK02G (Supplementary Tables 5 and 6). These findings strongly suggest that the expansion of B cells with IGHV4–28/IGHJ4-carrying IgG BCR may be the result of antigenic pressure leading to somatic hypermutation of CDR3.

**Table 2:** Somatic hypermutation in the CDR3 region of IGHV4–28/IGHJ4-carrying IgG-BCR in patient AHK03G.

Clone number	IGHV	IGHD	IGHJ	CDR3*	Number of reads
34	IGHV4–28	IGHD6–19	IGHJ4	CARKGSGWDGFDFW	8
48	IGHV4–28,IGHV4–34	IGHD6–19	IGHJ4	CARKGSGWDGFDFW	1
33	IGHV4–28,IGHV4–59,IGHV4-4	IGHD6–19	IGHJ4	CARKGSGWDGFDFW	115
36	IGHV4–28,IGHV4–59,IGHV4-4	IGHD6–19	IGHJ4	CARKGSGWDGFDFW	5
38	IGHV4–28,IGHV4–59,IGHV4-4	x	IGHJ4	CARKGSGWDGFDFW	2
37	IGHV4–28,IGHV4–59,IGHV4-4	IGHD6–19	IGHJ4	CARKGSGWDGCDFW	2
39	IGHV4–28,IGHV4–59,IGHV4-4	IGHD6–19	IGHJ4	CARKGSGWDGFAFW	2
40	IGHV4–28,IGHV4–59,IGHV4-4	x	IGHJ4	CARKGRGWDGFDFW	2
49	IGHV4–28,IGHV4–59,IGHV4-4	IGHD6–19	IGHJ4	CARKGSGWDGFDCW	1
50	IGHV4–28,IGHV4–59,IGHV4-4	IGHD6–19	IGHJ4	CARKGSGWDGLDFW	1
51	IGHV4–28,IGHV4–59,IGHV4-4	IGHD6–19	IGHJ4	CARKGSGWDRFDFW	1
52	IGHV4–28,IGHV4–59,IGHV4-4	IGHD6–19	IGHJ4	CARKGSGWEGFDYW	1
53	IGHV4–28,IGHV4–59,IGHV4-4	IGHD6–19	IGHJ4	CGRKGSGWDGFDFW	1
54	IGHV4–28,IGHV4–59,IGHV4-4	IGHD6–19	IGHJ4	CVRKGSGWDGFDFW	1
55	IGHV4–28,IGHV4–59,IGHV4-4	x	IGHJ4	CARKGSDWDGFDFW	1
56	IGHV4–28,IGHV4–59,IGHV4-4	x	IGHJ4	CARKGSGGDGFDFW	1
57	IGHV4–28,IGHV4–59,IGHV4-4	x	IGHJ4	CARKVSGWDGFDFW	1
58	IGHV4–28,IGHV4–59,IGHV4-4	IGHD6–19	IGHJ4	CVRKGSGWDGFDFW	1
61	IGHV4–28,IGHV4–59,IGHV4-4,IGHV4–34	IGHD6–19	IGHJ4	CARKGSGWDGFDSW	1
35	IGHV4–28,IGHV4–59,IGHV4-4,IGHV4–39	IGHD6–19	IGHJ4	CARKGSGWDGFDFW	6
63	IGHV4–28,IGHV4–59,IGHV4-4,IGHV4–39	IGHD6–19	IGHJ4	CARKGSGWDGFDFW	1

*The amino acid sequences that were different from that of clone number 34 are underlined.

### Detection of somatic hypermutation in the CDR3 region of IgG-BCR following vaccination

To validate the BCR repertoire analysis in terms of detecting the clonal proliferation of B cells and accompanying somatic hypermutation after antigenic stimulation, we investigated the IGH repertoire following the vaccination of seasonal influenza in healthy volunteer donors. Blood samples were taken on days 0, 7, and 28 following vaccination with the sampling schedule determined according to previous reports^12,20^. Wu *et al*. reported that the influenza vaccine induced changes in the IGHV repertoire in human peripheral blood by day 7. Thus, we selected IGHV subfamilies that showed a transient increase on day 7 following influenza vaccination in healthy donors. The healthy donor AHK13G had increased usage of IGHV1–18 and IGHV3–15 segments (Supplementary Fig. 1). High-throughput sequencing of IGH enabled us to identify the clonal expansion of the previously present B-cell clone carrying IGHV1–18/IGHJ5 following vaccination, and clonal expansion was accompanied by somatic hypermutation of the CDR3 region (Table 3). The expanded B-cell clones decreased by day 28. We also confirmed a transient clonal proliferation of B cells carrying IGHV3–15/IGHJ4 accompanied by somatic hypermutation of CDR3 (data not shown). The similar results were confirmed in another healthy volunteer donor AHK12G who received flu vaccination (Supplementary Table 7). However, we could not determine whether an oligoclonal expansion of B-cell clones accompanied by multiple related clones following flu vaccination was just the result of an expansion of pre-existing clones or a clonal response to flu vaccination accompanying affinity maturation. These findings demonstrate that high-throughput BCR repertoire analysis can identify antigen-driven clonal proliferation of B cells, although we do not show antigen specificity of the expanded B cells.

**Table 3:** Somatic hypermutation in the CDR3 region of IGHV1–28/IGHJ5-carrying IgG BCR in control donor AHK13G receiving flu vaccination.

IGHV	IGHD	IGHJ	CDR3*	Before	Day 7	Day 28
IGHV1–18	IGHD3–3	IGHJ5	CARQNDPYYDVWSGLGSNWLDPW	1	1664	102
IGHV1–18	IGHD3–3	IGHJ5	GARQNDPYYDVWSGLGSNWLDPW	0	21	2
IGHV1–18	IGHD3–3	IGHJ5	CARQNDPYDDVWSGLGSNWLDPW	0	20	2
IGHV1–18	IGHD3–3	IGHJ5	CARQNDPDYDVWSGLGSNWLDPW	0	19	1
IGHV1–18	IGHD3–3	IGHJ5	CARLNDPYYDFWTGLGSNWFDPW	0	19	0
IGHV1–18	IGHD3–3	IGHJ5	WARQNDPYYDVWSGLGSNWLDPW	0	16	3
IGHV1–18	IGHD3–3	IGHJ5	CARQNDPYCDVWSGLGSNWLDPW	0	15	0
IGHV1–18	IGHD3–3	IGHJ5	CARQNDPYYEVWSGLGSNWLDPW	0	10	3
IGHV1–18	IGHD3–3	IGHJ5	CARQNDPYSDVWSGLGSNWLDPW	0	9	1
IGHV1–18	IGHD3–3	IGHJ5	CARQKDPYYDVWSGLGSNWLDPW	0	9	0
IGHV1–18	IGHD3–3	IGHJ5	CARQNDPYYDVWSGLGSKWLDPW	0	8	0
IGHV1–18	IGHD3–3	IGHJ5	CAGQNDPYYDVWSGLGSNWLDPW	0	6	1
IGHV1–18	IGHD3–3	IGHJ5	CARQNDPYYDVWRGLGSNWLDPW	0	6	1
IGHV1–18	IGHD3–3	IGHJ5	CARLNDPYYDVWSGLGSNWLDPW	0	6	0
IGHV1–18	IGHD3–3	IGHJ5	CARQNDPYYDGWSGLGSNWLDPW	0	6	0
IGHV1–18	IGHD3–3	IGHJ5	CARQNDPYYDVGSGLGSNWLDPW	0	6	0
IGHV1–18	IGHD3–3	IGHJ5	CARQNDPYYAVWSGLGSNWLDPW	0	4	1
IGHV1–18	IGHD3–3	IGHJ5	CARQNDPYYDVWSGLGSNWFDPW	0	4	1
IGHV1–18	IGHD3–3	IGHJ5	CARQNDPYYDVWSGLGSNWRDPW	0	4	1
IGHV1–18	IGHD3–3	IGHJ5	FARQNDPYYDVWSGLGSNWLDPW	0	4	1
IGHV1–18	IGHD3–3	IGHJ5	CARQNDPCYDVWSGLGSNWLDPW	0	4	0
IGHV1–18	IGHD3–3	IGHJ5	CARQNDPSYDVWSGLGSNWLDPW	0	4	0
IGHV1–18	IGHD3–3	IGHJ5	CARQNDPYYDFWSGLGSNWLDPW	0	4	0
IGHV1–18	IGHD3–3	IGHJ5	CARQNDPYYDVWGGLGSNWLDPW	0	4	0
IGHV1–18	IGHD3–3	IGHJ5	CARQNDPYYYVWSGLGSNWLDPW	0	4	0

*The amino acid sequences that were different from the most prevalent clone are underlined. The related clones with less than 4 reads on day 7 were not shown.

## Discussion

We found preferential usage of the rearranged IGHV4–28/IGHJ4 gene in circulating IgG B cells in ITP. Oligoclonal proliferation of B cells has previously been reported, but the structural information of BCRs in primary ITP was unclear^21^. Roark *et al*. previously reported the usage of IGHV3–30 by the anti-platelet immunoglobulin from two patients with ITP using Fab/phage display libraries^22^. We did not find a difference in the usage of IGHV3–30 between ITP patients and control donors (Fig. 1). These conflicting results may be explained by the limited number of patients examined or different HLA backgrounds. Although the antigen specificity of BCRs encoded by the IGHV4–28/IGHJ4 gene in the present patient cohort remains to be elucidated, our findings may contribute to the development of genetic testing for the diagnosis of ITP.

Kuwana *et al*. previously reported that the mean number of anti-GP IIb/IIIa antibody-secreting B cells in peripheral blood was 8.2 cells per 10^5^ mononuclear cells in primary ITP patients. We detected 0.053% IGHV4–28-bearing B cells on average from the total population of IgG-expressing B cells in ITP. This figure is much higher than the frequency of anti-GP IIb/IIIa antibody-secreting B cells in blood mononuclear cells, but this may be explained by the fact that our assay was based on RNA expression and not on the size of the clones. Another possible explanation could be that the anti-GP IIb/IIIa antibody represents the pathogenic autoantibodies in ITP.

We found a skewed distribution of CDR3 lengths in IGHV4–28-carrying BCRs in ITP, and glycine and lysine were more frequently used in the short CDR3 regions. In general, glycine is found at the surface of proteins with loop regions and does not have a side chain, thereby providing high flexibility to the polypeptide chains at these locations. Lysine is a positively charged amino acid. This amino acid profile may reflect antigenic selection and affinity maturation.

The CDR regions are generally responsible for antigen recognition of immunoglobulins. In addition, some non-CDR regions are also responsible for antigen binding of antibodies^23^. Some residues of framework regions may comprise a part of the binding site, and others may provide structural support to CDR regions, thereby affecting antigen binding. We could not find any public clones using an IGHV4–28 segment. Our data have encouraged us to test the hypothesis that preferential usage of the rearranged IGHV4–28/IGHJ4 gene by circulating IgG-expressing B cells may be a potential diagnostic marker for primary ITP using a large patient cohort.

Limitation of this study could be that it remains unclear what the relevance of the usage of the rearranged IGHV4–28/IGHJ4 gene is for the pathogenesis of primary ITP in adults. One potential mechanism may be age because long-term antigenic stimulation may shape BCR repertoire. However, this explanation does not seem likely because there was no significant difference in age distribution between the patient cohort and control groups. The second potential explanation may be that the IGHV4–28 segment is a non-core gene, which is seen in some but not all individuals^24^. However, this possibility is also unlikely because all nine control donors and ITP patients had IGHV4–28-bearing B cells. Developing anti-idiotype antibody for the immunoglobulins reacting with platelets may solve this missing link between the frequent usage of the rearranged IGHV4–28/IGHJ4 and immune pathophysiology of ITP. There are also some technical limitations in this study. We could not differentiate the CDR3 sequences in small numbers of reads from a PCR error. This issue needs to be solved by another technology in the near future.

Some IGHV genes are expressed less frequently and the IGHV4–28 gene is one of those less frequently used genes^24^. The new BCR repertoire analysis described here comprised of adaptor-ligation PCR and next-generation sequencing could detect the oligoclonal expansions of B cells with small clonal sizes. Thanks to this method, we were able to detect the increased expression of this noncore gene by circulating B cells in ITP patients. To our knowledge, employing this strategy to characterize the immune receptor repertoires in ITP patients has never been reported.

The immunophenotype of circulating B lymphocytes and immunoglobulin isotype usage and antigen specificity of autoantibodies in ITP patients are crucial. In fact, several groups have previously addressed these questions. Li *et al*. reported that the numbers of IL-10-producing CD19 + CD24^hi^CD38^hi^ regulatory B cells were decreased in ITP patients^25^, and this was confirmed by another study group^26^. Exploration of immonophenotypes, cytokine production and antigen-specificity of IGHV4–28/IGHJ4-carrying B cells needs to be addressed in the future.

The IGHV4–28/IGHJ4-carrying clones mostly used an IGHG1 or IGHG2 gene segment as constant regions. Human IgG is divided into 4 subclasses with different heavy chains and each of them has its own functional properties. The IgG1 is the most prominent immunoglobulin in human sera and the IgG2 generally comprises 20 to 25% of total IgG. In human, IgG2 dominates in response to thymus-independent antigens such as pneumococcal polysaccharides^27^. Thymus-independent antigens can activate splenic marginal zone B cells. With regard to the IgG subclasses of anti-platelet autoantibodies, Chen *et al*. have previously reported that IgG1 was the most common isotype for platelet-associated autoantibodies directed against glycoprotein IIb/IIIa either alone or with other IgG subclass antibodies^28^. They also observed that some ITP patients had only IgG2 autoantibodies against IIb/IIIa. Taken together, ITP appears to be a heterogeneous disorder caused by anti-platelet autoantibodies that include a various patterns of IgG subclasses. Therefore, both thymus-dependent and thymus-independent antigens could contribute to the expansion of IGHV4–28/IGHJ4-carrying B cells in the present patient cohort.

In summary, we found that circulating IgG-expressing B cells preferentially used transcripts of a rearranged IGHV4–28/IGHJ4 gene in primary ITP, and that clonal expansion of IGHV4–28/IGHJ4-carrying B cells was accompanied by the somatic hypermutation in the CDR3 region. Although the responsible antigens remain unknown, these findings suggest that the expansion of IGHV4–28/IGHJ4-carrying B cells might be the result of antigenic pressure in ITP.

## Methods

### Patients

Eleven adult chronic ITP patients and nine volunteer donors without any hematologic disorders were included. All patients received a bone marrow examination. Diagnostic criteria for ITP were based on the American Society of Hematology (ASH) and the International Working Group (IWG) guidelines, and secondary ITP was excluded^5,29,30^. Patient characteristics are described in Supplementary Table 1. All control donors had received regular annual checkups and did not have any hematological diseases. Ages of control donors were 35 to 59 (median: 50.8) and seven female donors were included. There was no statistically significant difference in age between the ITP and control groups. No patients or control donors with hepatitis C virus or HIV infection were included^31,32^. Antibody tests for helicobacter pylori^33^ were positive in two ITP patients and two control donors. In some experiments, blood samples were collected from volunteer donors immunized with the 2016/2017 quadrivalent inactivated influenza vaccine (A/California/7/2009(H1N1)pdm09, A/Hong Kong/4801/2014(H3N2), B/Phuket/3073/2013(Yamagata lineage), B/Texas/2/2013(Victoria lineage)). These donors were invited to donate three peripheral blood samples: one before the vaccination (day 0), and the others on days 7 and 28 after the vaccination. This study was approved by the institutional review board of Akita University Graduate School of Medicine, and was performed in accordance with the Declaration of Helsinki. Written informed consent was obtained from the patients and healthy control donors before drawing blood samples.

### Samples and RNA extraction

Ten mL of heparinized peripheral blood was taken from each patient, and mononuclear cells (PBMCs) were isolated by density gradient centrifugation. Total RNA extraction and cDNA synthesis were performed by standard protocols. Total RNA was isolated from the PBMCs and purified with RNeasy Mini Kit (Qiagen, Hilden, Germany) according to the manufacturer’s instructions. The amount and purity of RNA were measured using the Agilent 2100 Bioanalyzer (Agilent Technologies, Palo Alto, CA, USA).

### Unbiased amplification and high-throughput sequencing of IgG-BCRs

Unbiased amplification of IgG-BCRs was performed by adaptor-ligation PCR, and the amplicons were sequenced by next-generation sequencing^19,34^. One hundred nanograms of total RNA was converted to cDNA. A specific primer BSL-18E containing polyT18 and a NotI site was used for cDNA synthesis^35,36^. Following the synthesis of dsDNA, a specific adaptor P10EA/P20EA was ligated to the blunted end of dsDNA, and then adaptor-ligated dsDNA was subjected to digestion with the NotI restriction enzyme. After clean up, PCR was performed with primer pairs specific for the constant region and the P20EA sequences. The second PCR was performed with a constant region-specific nested primer and a P20EA-specific primer. Primer sequences were previously reported^19^. After amplification, index sequences were added using a Nextera XT Index Kit v2 SetA (Illumina, San Diego, CA, USA). The indexed amplicons were mixed in an equimolar concentration, and the mixtures were subjected to next-generation sequencing using the Illumina Miseq paired-end platform (2 × 300 bp). Theoretically, total RNAs were extracted from approximately 10^5^ to 10^6^ B cells in each donor, although flow cytometric analysis was not routinely performed in this experiment. The sequencing depth was determined by the scale of samples and also in attempt to reduce the errors by too much deeper sequencing^37^. The cost of sequencing was an additional reason. Thus, the suitable sequencing depth was pre-determined as being approximately 100,000 reads per sample.

### Data analyses

Each sequence read was analyzed by the bioinformatics software created by Repertoire Genesis Incorporation (Ibaraki, Japan), and the usage of IGHV, IGHD, IGHJ, IGHC, and CDR3 (complementarity determining region 3) sequences were determined according to methods previously reported^19,34^. Briefly, the identification of V, D, J, and C regions were determined by identifying the sequence with the highest identity to reference sequence data sets available from the international ImMunoGeneTics information system (IMGT) database (http://www.imgt.org). The data processing, assignment, and data aggregation were automatically performed. The identical V, D, J, and deduced amino acid sequences of CDR3 were defined as a unique sequence read. A unique sequence read contained several variant sequences formed by somatic hypermutations, and thus we considered sequence reads sharing identical V, D, J segments and identical amino acid sequences of CDR3 as an identical clonal lineage. The Repertoire Genesis software automatically counted the numbers of unique sequence reads and ranked them in order of copy number.

### Repertoire diversity

To estimate the repertoire diversity, we calculated the indices of Shannon diversity, Simpson richness, and Pielou evenness using the R program^38^. The Shannon index was normalized by dividing with the logarithm of the total number of unique reads.

### Statistics

Statistical significance was tested with an independent sample t-test for comparing the mean values for two groups combined with the Levene’s test for equality of variances. Statistical analyses were performed using IBM SPSS version 23 software.

### Data Availability

For original data, please contact mhirokawa@hos.akita-u.ac.jp.

## Supplementary information

Supplementary information

Supplementary dataset 1
