# Supplementary material for: High-throughput sequencing of IgG B-cell receptors reveals frequent usage of the rearranged IGHV4–28/IGHJ4 gene in primary immune thrombocytopenia
Source: Sci Rep. 2019 Jun 14;9:8645. doi: 10.1038/s41598-019-45264-2 (PMC6570656; doi:10.1038/s41598-019-45264-2)
Supplement: Supplementary file 1 — Supplementary information [file 41598_2019_45264_MOESM1_ESM.docx]

**Supplementary information**

**High-throughput sequencing of IgG B-cell receptors reveals frequent usage of the rearranged IGHV4-28/IGHJ4 gene in primary immune thrombocytopenia**

Makoto Hirokawa, Naohito Fijishima, Masaru Togashi, Akiko Saga, Ayumi Omokawa, Tomoo Saga, Yuki Moritoki, Shigeharu Ueki, Naoto Takahashi, Kazutaka Kitaura, Ryuji Suzuki

**
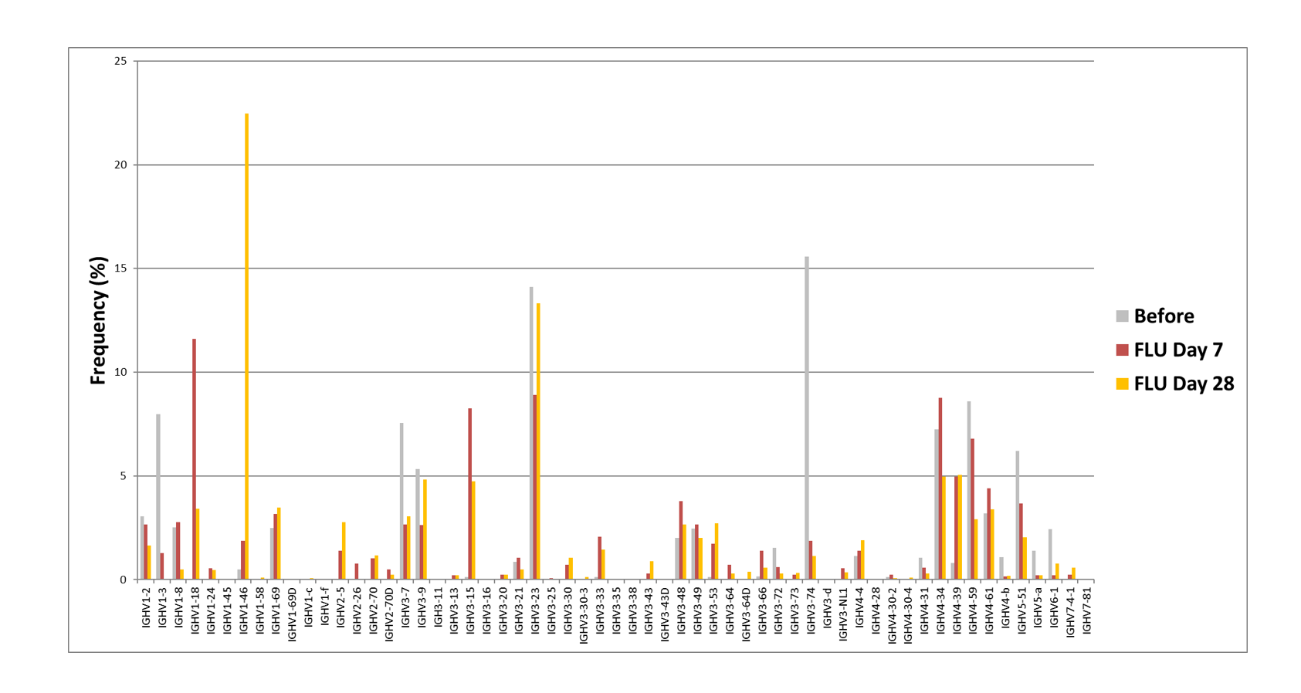
**

**Supplementary Figure 1. The IGHV repertoire of IgG BCRs following influenza vaccination in donor AHK13G.**

**Supplementary Table 1. Patient characteristics**

| Sample ID | Age/Sex | WBC | Hb | PLT  (x10^9^/L)* | Lym  (%) | Treatment | Previous treatment | Anti-platelet antibody testing** | Anti-H. pylori IgG*** |
| --- | --- | --- | --- | --- | --- | --- | --- | --- | --- |
| AHK01 | 30/F | 10800 | 12.5 | 80 | 23.9 | Prednisolone |  | Negative | Negative |
| AHK02 | 38/F | 7400 | 13 | 92 | 34.5 | None | Prednisolone, splenectomy | Positive | Negative |
| AHK03 | 74/F | 8200 | 12.3 | 153 | 42.5 | Cyclosporine | Prednisolone, splenectomy | Positive | Negative |
| AHK04 | 60/F | 6500 | 13.2 | 38 | 43.5 | Prednisolone |  | Negative | Negative |
| AHK06 | 47/F | 3600 | 14.3 | 71 | 50.6 | None |  | Negative | Negative |
| AHK07 | 55/F | 6700 | 15 | 42 | 18.0 | Prednisolone |  | Positive | Negative |
| AHK08 | 57/M | 6000 | 12.9 | 8 | 21.6 | Romiplostim | Prednisolone | Negative | Negative |
| AHK09 | 69/M | 4900 | 14.9 | 71 | 35.9 | None |  | Negative | Positive |
| AHK10 | 71/M | 5400 | 15.1 | 33 | 15.9 | Prednisolone |  | Negative | Negative |
| AHK011 | 80/F | 5500 | 13.3 | 100 | 23.4 | None | H. pylori eradication | Negative | Negative |
| AHK021 | 86/F | 6900 | 14.6 | 146 | 34.0 | None | Prednisolone | Negative | Positive |

* Platelet counts were the time when blood samples were taken for this experiment.

** Anti-platelet antibody was tested by the MPHA method. All control donors were negative for antiplatelet antibody testing.

*** Anti-helicobacter pylori IgG antibody was tested by the EIA method. Two control donors were positive for anti-helicobacter pylori IgG antibody.

**Supplementary Table 2. Distribution of the CDR3 length of IGHV4-28-carrying IgG-BCR in ITP and control donors**

|  | Control | ITP | Total |
| --- | --- | --- | --- |
| Numbers of clones | 34 | 186 | 220 |
| Mean | 18.7 a.a. | 16.5 a.a. | 16.8 a.a. |
| Median | 18.5 a.a. | 16.5 a.a. | 17.0 a.a. |
| Min | 8 a.a. | 11 a.a. | 8 a.a. |
| Max | 27 a.a. | 25 a.a. | 27 a.a. |
| Standard error | 0.694 | 0.241 | 0.236 |
| Kurtosis | 0.220 | 0.346 | -0.019 |
| Skewness | -0.335 | 0.953 | 0.722 |

**Supplementary Table 3. Usage of glycine (G) in the CDR3 region of IGHV4-28-carrying IgG BCR**

|  |  | Clones with respective numbers of glycine (G) residue | | | | | |  |
| --- | --- | --- | --- | --- | --- | --- | --- | --- |
|  | Total numbers of clones examined | 0 | 1 | 2 | 3 | 4 | 5 | P value  (chi-square) |
| Control | 34 | 3 | 9 | 13 | 5 | 2 | 2 |  |
| vs. ITP | 186 | 7 | 62 | 46 | 59 | 12 | 0 | 0.003 |
|  |  |  |  |  |  |  |  |  |
| Control | 34 | 3 | 9 | 13 | 5 | 2 | 2 |  |
| vs. ITP aa 13-14 | 70 | 1 | 9 | 10 | 44 | 6 | 0 | 0.000097 |
|  |  |  |  |  |  |  |  |  |
| ITP all length | 70 | 1 | 9 | 10 | 44 | 6 |  |  |
| vs. ITP aa 13-14 | 186 | 7 | 62 | 46 | 59 | 12 |  | 0.000092 |

**Supplementary Table 4. Usage of lysine (K) in the CDR3 region of IGHV4-28-carrying IgG BCR**

|  |  | Clones with respective numbers of lysine (K) residue | | | |
| --- | --- | --- | --- | --- | --- |
|  | Total numbers of clones examined | 0 | 1 | 2 | P value  (chi-square) |
| Control | 34 | 27 | 7 | 0 |  |
| vs. ITP | 186 | 120 | 39 | 27 | 0.053 |
|  |  |  |  |  |  |
| Control | 34 | 27 | 7 | 0 |  |
| vs. ITP aa 13-14 | 70 | 17 | 28 | 25 | 0.00000017275 |
|  |  |  |  |  |  |
| ITP all length | 186 | 120 | 39 | 27 |  |
| vs. ITP aa 13-14 | 70 | 17 | 28 | 25 | 0.000000048779 |

**Supplementary Table 5. Somatic hypermutation in the CDR3 region of IGHV4-28/IGHJ4-carrying IgG BCR in patient AHK07G**

| Clone number | IGHV | IGHD | IGHJ | CDR3* | Number  of reads |
| --- | --- | --- | --- | --- | --- |
| 95 | IGHV4-28,IGHV4-61,IGHV4-4 | x | IGHJ4 | CAKKGDGSALGYW | 20 |
| 96 | IGHV4-28 | x | IGHJ4 | CAKKGDGSALGYW | 3 |
| 109 | IGHV4-28,IGHV4-39,IGHV4-31 | x | IGHJ4 | CAKKGDGSALGYW | 1 |
| 118 | IGHV4-28,IGHV4-61,IGHV4-4 | x | IGHJ4 | CPKKGDGSALGYW | 1 |
| 124 | IGHV4-28,IGHV4-61,IGHV4-4,IGHV4-34 | x | IGHJ4 | CAEKGDGSALGYW | 1 |
| 107 | IGHV4-28,IGHV4-34,IGHV4-31 | x | IGHJ4 | CARKGDGSALGYW | 1 |
| 116 | IGHV4-28,IGHV4-61,IGHV4-4 | x | IGHJ4 | CAKKVDGSALGYW | 1 |
| 100 | IGHV4-28 | x | IGHJ4 | CAKKGEGSALGYW | 1 |
| 110 | IGHV4-28,IGHV4-61,IGHV4-4 | x | IGHJ4 | CAKKGDGAALGYW | 1 |
| 111 | IGHV4-28,IGHV4-61,IGHV4-4 | x | IGHJ4 | CAKKGDGEALGYW | 1 |
| 113 | IGHV4-28,IGHV4-61,IGHV4-4 | x | IGHJ4 | CAKKGDGSELGYW | 1 |
| 114 | IGHV4-28,IGHV4-61,IGHV4-4 | x | IGHJ4 | CAKKGDGSTLGYW | 1 |
| 97 | IGHV4-28,IGHV4-61,IGHV4-4 | x | IGHJ4 | CAKKGDGSARGYW | 2 |
| 112 | IGHV4-28,IGHV4-61,IGHV4-4 | x | IGHJ4 | CAKKGDGSALEYW | 1 |
| 108 | IGHV4-28,IGHV4-39 | x | IGHJ4 | CAKKGDGSALGNW | 1 |
| 120 | IGHV4-28,IGHV4-61,IGHV4-4 | x | IGHJ4 | FAKKGDGSALGYW | 1 |
| 121 | IGHV4-28,IGHV4-61,IGHV4-4 | x | IGHJ4 | GAKKGDGSALGYW | 1 |
| 103 | IGHV4-28 | x | IGHJ4 | VAKKGDGSALGYW | 1 |
| 98 | IGHV4-28,IGHV4-61,IGHV4-4 | x | IGHJ4 | WAKKGDGSALGYW | 2 |
| 104 | IGHV4-28 | x | IGHJ4 | WAKKGDGSALGYW | 1 |
| 125 | IGHV4-28,IGHV4-61,IGHV4-4,IGHV4-39 | x | IGHJ4 | WAKKGDGSALGYW | 1 |
| 115 | IGHV4-28,IGHV4-61,IGHV4-4 | x | IGHJ4 | CAKKGEGSTLGYW | 1 |
| 117 | IGHV4-28,IGHV4-61,IGHV4-4 | x | IGHJ4 | CGKKDDGSTMGYW | 1 |
| 119 | IGHV4-28,IGHV4-61,IGHV4-4 | x | IGHJ4 | CSKNGDGSALGYW | 1 |
| 122 | IGHV4-28,IGHV4-61,IGHV4-4 | x | IGHJ4 | GAKKGDGSARGYW | 1 |
| 102 | IGHV4-28 | x | IGHJ4 | GEKKGDGSALGYW | 1 |
| 123 | IGHV4-28,IGHV4-61,IGHV4-4 | x | IGHJ4 | WAKKGEGSALGYW | 1 |
| 105 | IGHV4-28 | x | IGHJ4 | WEKKGEGSALGYW | 1 |
| 101 | IGHV4-28 | x | IGHJ4 | CANKGDGWALGYW | 1 |

*The amino acid sequences that were different from that of the most prevalent clone are underlined.

**Supplementary Table 6. Somatic hypermutation in the CDR3 region of IGHV4-28/IGHJ4-carrying IgG BCR in patient AHK02G**

| Clone number | IGHV | IGHD | IGHJ | CDR3* | Read number |
| --- | --- | --- | --- | --- | --- |
| 8 | IGHV4-28,IGHV4-61 | x | IGHJ4 | CARIPPTTGTAHYFDQW | 17 |
| 9 | IGHV4-28 | x | IGHJ4 | CARIPPTTGTAHYFDQW | 5 |
| 12 | IGHV4-28,IGHV4-61,IGHV4-59,IGHV4-4 | x | IGHJ4 | CARIPPTTGTAHYFDQW | 2 |
| 30 | IGHV4-28,IGHV4-61 | x | IGHJ4 | CARSPPTTGTAHYFDQW | 1 |
| 13 | IGHV4-28 | x | IGHJ4 | CARIHPTTGTAHYFDQW | 1 |
| 14 | IGHV4-28 | x | IGHJ4 | CARIPPKTGTAHYFHQW | 1 |
| 31 | IGHV4-28,IGHV4-61,IGHV4-39 | x | IGHJ4 | CARIPPTTETAHYFDQW | 1 |
| 10 | IGHV4-28 | x | IGHJ4 | CARIPPTTGTDHYFDQW | 2 |
| 26 | IGHV4-28,IGHV4-61 | x | IGHJ4 | CARIPPTTGTSHYFDQW | 1 |
| 18 | IGHV4-28 | x | IGHJ4 | CARIPPTTGTTHYFDQW | 1 |
| 19 | IGHV4-28 | x | IGHJ4 | CARIPPTTGTVHYFDQW | 1 |
| 16 | IGHV4-28 | x | IGHJ4 | CARIPPTTGTAYYFDQW | 1 |
| 11 | IGHV4-28,IGHV4-61 | x | IGHJ4 | CARIPPTTGTAHYCDQW | 2 |
| 24 | IGHV4-28,IGHV4-61 | x | IGHJ4 | CARIPPTTGTAHYLDQW | 1 |
| 25 | IGHV4-28,IGHV4-61 | x | IGHJ4 | CARIPPTTGTAHYYDQW | 1 |
| 21 | IGHV4-28 | x | IGHJ5 | CARIPPTTGTAHYFDPW | 1 |
| 15 | IGHV4-28 | x | IGHJ4 | CARIPPNTGTAHYFDQW | 1 |
| 17 | IGHV4-28 | x | IGHJ4 | CARIPPTTGTSNYFDQW | 1 |
| 20 | IGHV4-28 | x | IGHJ4 | CARSPPTTGKAHYFDQW | 1 |
| 22 | IGHV4-28,IGHV4-61 | x | IGHJ4 | CARIPPTPGTAHDFDQW | 1 |
| 23 | IGHV4-28,IGHV4-61 | x | IGHJ4 | CARIPPTTGTAHDYDQW | 1 |
| 27 | IGHV4-28,IGHV4-61 | x | IGHJ4 | CARIPPTTWTTHYFDQW | 1 |
| 28 | IGHV4-28,IGHV4-61 | x | IGHJ4 | CARIPTTTGTDHYLDQW | 1 |
| 29 | IGHV4-28,IGHV4-61 | x | IGHJ4 | CARMPPTTGMAHYFDQW | 1 |
| 32 | IGHV4-28,IGHV4-39 | x | IGHJ4 | CARIPTTTGTANYFEQW | 1 |

*The amino acid sequences that were different from that of the most prevalent clone are underlined.

**Supplementary Table 7. Somatic hypermutation in the CDR3 region of IGHV3-23/IGHJ4-carrying IgG BCR in control donor AHK12G receiving flu vaccination**

| IGHV | IGHD | IGHJ | CDR3* | Before | Day 7 | Day 28 |
| --- | --- | --- | --- | --- | --- | --- |
| IGHV3-23 | x | IGHJ4 | CAKFVARPVADYFDYW | 1 | 690 | 1001 |
| IGHV3-23 | x | IGHJ4 | CAKFVARAVADHFDFW | 0 | 140 | 0 |
| IGHV3-23 | x | IGHJ4 | CAKFVTRPVADYFDYW | 0 | 3 | 0 |
| IGHV3-23 | x | IGHJ4 | CAKFVARPVADYFDSW | 0 | 3 | 0 |
| IGHV3-23 | x | IGHJ4 | CAKFGARPVADYFDYW | 0 | 2 | 0 |
| IGHV3-23 | x | IGHJ4 | CAKFVAGPVADYFDYW | 0 | 2 | 0 |
| IGHV3-23 | x | IGHJ4 | CAKFVASPVADYFDYW | 0 | 2 | 0 |
| IGHV3-23 | x | IGHJ4 | CAKFVARPMADYFDYW | 0 | 2 | 0 |
| IGHV3-23 | x | IGHJ4 | CAKFVARPVTDYFDYW | 0 | 2 | 1 |
| IGHV3-23 | x | IGHJ4 | CAKFVARPVADYFGYW | 0 | 2 | 3 |
| IGHV3-23 | x | IGHJ4 | CAKFVARPVADYFDCW | 0 | 2 | 0 |

*The amino acid sequences that were different from the most prevalent clone are underlined. The related clones with less than 2 reads on day 7 were not shown.
